# Preliminary Study of the Influence of Red Blood Cells Size on the Determinism of the Breed in Cattle

**DOI:** 10.1155/2014/429495

**Published:** 2014-02-06

**Authors:** Nezar Adili, Mohamed Melizi, Hadj Belabbas, Abdelhamid Achouri

**Affiliations:** ^1^Department of Veterinary Medicine, University of El-Hadj Lakhdar, 05000 Batna, Algeria; ^2^Department of Microbiology and Biochemistry, University of Mohamed BOUDIAF, 28000 M'sila, Algeria; ^3^Department of Veterinary Medicine, University of Mohamed Cherif Messaadia, 41000 Souk Ahras, Algeria

## Abstract

This study was carried out on five cattle groups, local, cross, Prim'Holstein, Montbeliard, and Brown of the Alps, in order to study the influence of breed on erythrocytes diameter. For each breed, blood samples were taken from 15 adult females by jugular venipuncture; blood smears were made on slides immediately after the blood collection and stained according to the method of May-Gründwald Giemsa. Morphometric study was realized using the OPTIKA Pro Vision software. The statistical analysis was assessed by using the descriptive boxplots test and ANOVA. The size of red blood cells is greater in the imported Brown of the Alps breed (5,32 ± 0,19) and also in our local breed (5,23 ± 0,10), whereas they were smaller in the Montbeliard breed (4,79 ± 0,21). This investigation allowed us to show that from a drop of blood we can have an idea of the bovine breeds, taking into account the size of the erythrocytes.

## 1. Introduction

The mature red blood cell (RBC) of the adult bovine is biconcave in shape [1, 2], has a width of 5-6 *μ*m, and has minimal central pallor and relatively long lifespan of approximately 130 days [3]. Red blood cell shape is relatively uniform; polychromatophils are generally absent from the blood of adult cattle [1, 3]. Age, sex, exercise, and emotional state are variables to be considered when establishing references values in domestic cattle [4–10]. Bulls have appreciably greater red blood cells counts than cows; breed differences have been reported for beef cattle, which have greater red blood cells values than dairy cattle breeds; lactating cows have consistently lower red blood cells values than nonlactating cows [1, 3, 9]; altitude polyglobulia is well documented and extensively studied [9, 11], while few references were found on blood cell morphometry. This study was realized on local, imported, and crossed cattle, in order to investigate the influence of breed on red blood cell size; its aims is to develop criteria selection of breeds, taking into account the morphometry of red blood cells.

## 2. Materials and Methods

### 2.1. Region of the Study and Animals

This study was conducted in the region of Batna, located in the eastern part of Algeria, 410 km from the capital city Algiers at an altitude of about 1000 m. For the realization of sampling we selected 15 nonpregnant adult females (3–5 years old) per breed, in order to eliminate the effect of age, sex, and pregnancy. All the cattle were clinically healthy, free from internal and external parasites, and belonging to the following breeds:

- local brown of Atlas breed;
- crossing breed (local and imported);
- three imported breeds: Prim'Holstein (Pie-Red), Montbeliard, and Brown of the Alps.

### 2.2. Blood Samples and Smears

After disinfecting of the sampling area, blood samples were taken from the jugular vein [12]; smears were confectioned on microscope slides just after venipuncture without anticoagulants which may interfere and induce some cytoplasmic and morphometric cell changes and on the extreme provoke degranulation of some blood cells [13–15]. Slides are precisely identified (order number, breed) and classified in slides racks which are equipped with an information sheet including breed and age of the animals following the order number mentioned on the slide; these pieces of information are recorded immediately after each sampling and smear realization.

### 2.3. Blood Smears Staining

Staining of blood smears was carried out following the classical mixed panoptic staining of Pappenheim, especially by the dye of May-Gründwald Giemsa (M.G.G, Romanowsky type) the best and most appropriate staining to mark the mammalians erythrocytes, respecting always the protocol cited by Ledieu [12], Gabe [16], and Houwen [17].

### 2.4. Morphometric Study

For several and even until the last years, morphometric studies of red blood cells are essentially based on linear measures of erythrocytes size. Using an ocular micrometer and an objective micrometer is the only valid and recognized method to measure the size of erythrocytes. The diameter of the red blood cells is measured or estimated roughly under an optical microscope at a magnification immersion (×100) [18–20].

In view of the exponential technological progress and, besides the tedious and monotonous use of the ocular micrometer, make the use of the instrument in question disappearing especially with the advent and, the generalization of high performance microscopes.

In our study, we used a high-performance professional optical microscope: OPTIKA B-350 (Ver.4.0.0); it is a modern, ergonomic, binocular microscope equipped with a digital camera of high resolution OPTIKAM (Ver.4.1.0) enabling the display of the microscopic image of the smear placed in the microscope on a computer in real time. The morphometric study of red blood cells is performed with special OPTIKA Pro Vision software of the OPTIKA microscope; measurement operations of this software are the digital version of the more traditional techniques of morphometry with optical microscopes. This is an integral and ergonomic software, which in addition to the functions of image capture and camera control allows efficient processing and analysis of microscopic images in an easy manner for any type of application and research.

Before starting the actual morphometric study, it is necessary to scan the microscopic images of red blood cells. The taking of photos for all subjects of different breeds is an essential step; it allows fixing the microscopic images observed and recording so that you can manipulate them using the software.

The information concerning morphometric data of red blood cells is accurate only if a scale has been correctly entered and well calibrated, which requires scanning the micrometer scale engraved on the micrometric slide on the immersion objective (×100).

To study the influence of breed on the RBC size in cattle, we measured for each female the diameter of 50 red blood cells, and then we determine the average for all the cattle breeds. The morphometric study of red blood cells was performed by always respecting the guidelines and instructions of the manufacturer of the software.

### 2.5. Statistical Analysis

To compare the experimental data, descriptive statistics by boxplots were realized using the Microsoft Office Excel 2007. Analysis of variances (ANOVA) was also performed with the MedCalc statistical software (version 12.7, Copyright © 1993–2013 Med Clack software bvba); *P* value under 0,05 was considered statistically significant.

## 3. Results and Discussion

**Figure 1:**
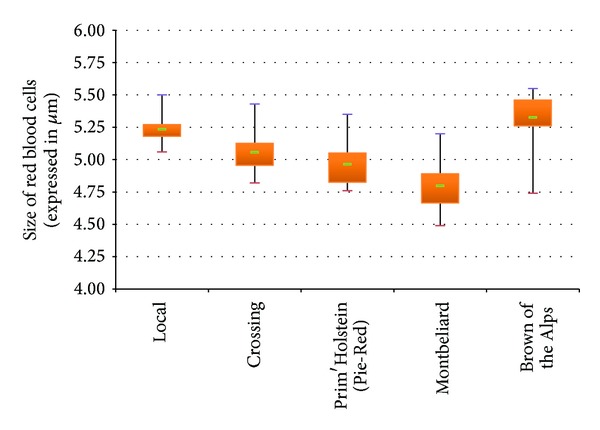
Influence of breed on the diameter of red blood cells in cattle.

In Figure 1 we presented the influence of breed on the RBC size. Firstly, no overlap was detected between the group of the Brown of the Alps with the groups of crossed cattle, the Prim'Holstein, and the Montbeliard; it is the same for the group of local cattle with the crossed, Prim'Holstein, and the Montbeliard groups. On the other hand, we found the presence of a slight overlap between the Brown of the Alps and local breed and also between the boxes of crossed, Prim'Holstein, and the Montbeliard cattle. We should note that the average of the Brown of the Alps group is clearly higher than the other groups, followed respectively by the local and then the crossed and the Prim'Holstein, whereas it is the lowest for the Montbeliard, which tends to indicate a significant influence of the breed factor. Statistical analysis done with ANOVA shown in Table 1, confirms major significant differences between the five breeds (*P* < 0,001).

According to our results, it seems that the breed can affect the diameter of erythrocytes in cattle; the values obtained are still within the range of international standards cited by different authors [1, 3, 9].

In addition, we have to note that the classical measures of red blood cells diameter, with the ocular micrometer, are quite difficult and imprecise. Measures were done with the software cited above, allowing performing quite correct comparative studies in limiting the human factor involved in the measurements realized with the ocular micrometer by choosing the best place. In addition, this new measurement method is very simple, direct, easy to make, and not much expensive.

However, this investigation needs to be complemented by studies on the influence of breed on the circumference and the surface of red blood cells, not only in cattle but also for all pets.

Circumference and surface measures of red blood cells appear to be more appropriate for comparative studies; moreover, it seems that these two parameters are more representative on the research of morphological changes in the morphometry of red blood cells.

## 4. Conclusion

This preliminary study on the influence of breed on red blood cell diameter in cattle, realized on 75 blood smears, has shown the following.

- The size of red blood cells is greater in the imported Brown of the Alps breed and in our local breed, whereas the Montbeliard breed cattle have smaller red blood cells.
- It turns out that the morphometric studies of erythrocytes made by this type of software are more appropriate and more accurate than measurements with the ocular micrometer.
- Other studies on the measurements of the circumference and the surface of red blood cells by this type of software seem necessary, in order to investigate the exact influence of breed on red blood cell morphometry.

Finally, through this study we can conclude that from a drop of blood we can have an idea about the cattle breeds taking into account the size or the diameter of the erythrocytes.

## Figures and Tables

**Table 1:** Terminal values, averages, standard deviation, and quartiles of the diameter of red blood cells of cattle (expressed in *μ*m).

Breeds (*N* = 15)	Min. val.	*Q*1	M & s.d	*Q*3	Max. val.
Local	5,06	5,18	5,23 ± 0,10	5,27	5,50
Crossing	4,82	4,96	5,05 ± 0,16	5,13	5,43
Prim'Holstein (Pie-Red)	4,76	4,83	4,96 ± 0,16	5,05	5,35
Montbeliard	4,49	4,67	4,79 ± 0,21	4,89	5,20
Brown of the Alps	4,74	5,26	5,32 ± 0,19	5,46	5,55

(Min. val.: minimal value; Q1: first quartile; M & s.d: mean and standard deviation; Q3: third quartile; Max. val.: maximal value).
